# Discovery of a major QTL for root-knot nematode (*Meloidogyne incognita*) resistance in cultivated sweetpotato (*Ipomoea batatas*)

**DOI:** 10.1007/s00122-021-03797-z

**Published:** 2021-04-03

**Authors:** Bonny Michael Oloka, Guilherme da Silva Pereira, Victor A. Amankwaah, Marcelo Mollinari, Kenneth V. Pecota, Benard Yada, Bode A. Olukolu, Zhao-Bang Zeng, G. Craig Yencho

**Affiliations:** 1 Department of Horticultural Science, North Carolina State University, 214 Kilgore Hall, Box 7609, Raleigh, NC 27695 USA; 2 National Agricultural Research Organisation (NARO), National Crops Resources Research Institute (NaCRRI), Namulonge, P.O. Box 7084, Kampala, Uganda; 3 International Potato Center (CIP), Nairobi, Kenya; 4 CSIR-Crops Research Institute, Kumasi, Ghana; 5 University of Tennessee, Knoxville, TN 37996 USA

## Abstract

**Key message:**

Utilizing a high-density integrated genetic linkage map of hexaploid sweetpotato, we discovered a major dominant QTL for root-knot nematode (RKN) resistance and modeled its effects. This discovery is useful for development of a modern sweetpotato breeding program that utilizes marker-assisted selection and genomic selection approaches for faster genetic gain of RKN resistance.

**Abstract:**

The root-knot nematode [Meloidogyne incognita (Kofoid & White) Chitwood] (RKN) causes significant storage root quality reduction and yields losses in cultivated sweetpotato [Ipomoea batatas (L.) Lam.]. In this study, resistance to RKN was examined in a mapping population consisting of 244 progenies derived from a cross (TB) between ‘Tanzania,’ a predominant African landrace cultivar with resistance to RKN, and ‘Beauregard,’ an RKN susceptible major cultivar in the USA. We performed quantitative trait loci (QTL) analysis using a random-effect QTL mapping model on the TB genetic map. An RKN bioassay incorporating potted cuttings of each genotype was conducted in the greenhouse and replicated five times over a period of 10 weeks. For each replication, each genotype was inoculated with ca. 20,000 RKN eggs, and root-knot galls were counted ~62 days after inoculation. Resistance to RKN in the progeny was highly skewed toward the resistant parent, exhibiting medium to high levels of resistance. We identified one major QTL on linkage group 7, dominant in nature, which explained 58.3% of the phenotypic variation in RKN counts. This work represents a significant step forward in our understanding of the genetic architecture of RKN resistance and sets the stage for future utilization of genomics-assisted breeding in sweetpotato breeding programs.

**Supplementary Information:**

The online version contains supplementary material available at (10.1007/s00122-021-03797-z).

## Introduction

Plant parasitic nematodes are major pathogens of many cultivated crops (Agrios 2004). The root-knot nematode (RKN), *Meloidogyne incognita*, is a widely distributed plant parasitic nematode and is responsible for billions of dollars in crop losses annually (Sasser and Freckman 1987; Cervantes-Flores et al. 2008b). Root-knot nematodes (*Meloidogyne* spp.) are distributed worldwide and the damage they inflict on the production of field crops is estimated at about 10% worldwide (Whitehead 1998). In eastern Africa, RKNs are reported to affect sweetpotato (Namaganda et al. 1993; Makumbi-Kidza et al. 2000; Karuri et al. 2017), cassava and banana (Coyne et al. 2006). However, much of the damage by RKNs goes undetected due to their associations with fungi and bacteria in disease complexes (Cervantes-Flores et al. 2008b). The cracking and secondary infections reduces the market value of the sweetpotato storage roots by directly affecting their quality.

RKNs are obligatory sedentary endoparasites with a life cycle of one to two months. Embryos develop and hatch as second-stage larvae (L2) that move through the soil and invade the plant root (Sasser 1980). Upon infection, the larvae establish a feeding site within the root cortex and they undergo three additional molts before transitioning to an adult nematode. *M. incognita* is a mitotic parthenogenetic species that produces a continuous infection chain once established. The feeding sites appear as rounded galls (knots) on the root vascular tissue of infected plants, thus disrupting their capacity to uptake nutrients from the soil. On fleshy storage roots of sweetpotato, the infection can appear as cracks, although these are also associated with various other environmental factors like soil texture and moisture (Lawrence et al. 1986). In their research on RKNs, Lawrence et al. (1986) suggested that pathogens may be predisposing the roots to cracking, rather than RKN a causal factor. The suggestion was based on a lack of correlation between number of cracked roots and initial RKN population size. They observed that when rainfall was more uniform, the storage roots did not crack although nematodes were present in them (Lawrence et al. 1986). There is also a genetic component to this since some genotypes crack more often than others when grown in warm, wet and sandy soils.

Managing root-knot nematodes has often involved the use of neurotoxic nematicides in combination with cultural practices. Besides the obvious health and environmental risk that nematicides pose, their cost is prohibitive to small scale farmers and growers of sweetpotato (Gasapin 1984). The safest, most sustainable and economic control method is the use of resistant plant genotypes. Currently, most popular and widely grown sweetpotato cultivars are highly susceptible to *M. incognita* (Kofoid & White) Chitwood and *M. javanica* (Kofoid & White) Chitwood (Cervantes-Flores et al. 2002). On a global scale, M. incognita is the most important and most widely distributed nematode species that affect cultivated sweetpotato (Jatala 1991). It prefers warm temperatures for completion of its lifecycle, rapidly increasing in number by undergoing 4 to 5 generations per growing season (Cervantes-Flores 2000). The RKN species, *M. javanica*, is predominant in southern U.S but is not globally distributed (Cervantes-Flores et al. 2002).

The genetic basis and plant factors mediating sweetpotato resistance to RKN are not well understood. Ukoskit et al. (1997) hypothesized single-gene qualitative resistance, whereas quantitative resistance has been hypothesized by several other researchers (Cordner et al. 1954; Giamalva et al. 1961; Jones and Dukes 1980; Cervantes-Flores et al. 2008b). Jones and Dukes (1980) suggested that independent sources of resistance to different strains of RKN were responsible for the observed differences in inheritance and that the genes originated from multiple origins (Mcharo et al. 2005). The RKN species *M. Incognita* has 4 different physiological races i.e. race 1, 2, 3 and 4, with race 3 being the most predominant in North Carolina. Histological studies have shown that RKNs in the juvenile (L2) stage penetrate both susceptible and resistant sweetpotato clones, as well as other *Ipomoea* species (Komiyama et al. 2006). However, Komiyama et al (2006) observed that localized necrotic reactions prevent further pathogen development in resistant clones, whereas in susceptible genotypes, the pathogen is able to establish itself. This view agrees with observations made by earlier researchers (Dropkin 1969; Paulson and Webster 1972). Nematodes that fail to establish feeding sites have been observed to either die or leave the roots (Koyimana et al. 2006). It has also been noted that resistance to *M. incognita* occurs via a hypersensitive reaction in sweetpotato (Dean and Struble 1953; Gentile et al. 1962; Martin and Birchfield 1973; Jones and Dukes 1980) and in other crops (Okamoto and Mitsui 1974).

Molecular markers have been widely used in many crops to identify and map genes associated with resistance to nematodes (Barr et al. 1998; Wang et al. 2001; Ynturi et al. 2006), but very few of such studies have been successfully conducted in sweetpotato (Ukoskit et al. 1997; Mcharo et al. 2005; Cervantes-Flores et al. 2008b; Nakayama et al. 2012). Ukoskit et al. (1997) used random amplified polymorphic DNA (RAPD) markers in a population of 71 individuals derived from a cross between ‘Regal’ (resistant) and ‘Vardaman’ (susceptible), and the marker OP15_1500_ was weakly associated (*P* = 0.037) with RKN resistance in the cross. Mcharo et al. (2005) employed amplified fragment length polymorphism (AFLP) markers genotyping on two unrelated sweetpotato populations and applied logistic regression and discriminant analysis to study RKN resistance. They report the ability to predict and classify the phenotype with an accuracy of 88.75 and 88.04%. However, since their population sizes were small (48 half sibs and 54 full sibs) they could have overestimated the effect of the markers (Cervantes-Flores et al. 2008b). Using AFLP markers, which required the construction of two separate parental maps, Cervantes-Flores et al. (2008b) hypothesized that resistance to RKN was conferred by several genes in the ‘Tanzania’×‘Beauregard’ cross studied in this research. They detected nine QTL associated with RKN resistance, each of which showed a relatively small genetic effect. They also identified three unmapped duplex markers that explained most of the phenotypic variation (~ 45%), but they were not able to map those particular markers within the parental linkage maps. They concluded that the higher-dose markers would be more informative when placed on the genetic map, and that they were potentially associated with one or two major genes (Cervantes-Flores et al. 2008b). For hexaploid sweetpotato, high dose markers are those with more than 2 reference alleles (duplex to hexaplex) on a given locus whereas low dose markers are those with 0 (nulliplex), or 1 (simplex) reference allele on the marker locus. More recently, Nakayama et al. (2012) have conducted an analysis of resistance to multiple races of southern root-knot nematode (SRKN). They suggested that race-specific resistance is more likely conferred by single genes and that the genes for resistance against each race are closely located (Nakayama et al. 2012). However, like Ukoskit et al. (1997), their population of 86 F1 progeny was small, and they therefore could have overestimated the marker effects.

In this work, we describe the use of single- and multiple-dose bi-allelic single-nucleotide polymorphism (SNP) and insertion–deletion (indel) markers on the ‘Tanzania’×‘Beauregard’ (TB) genetic linkage map to identify QTL for the RKN, *Meloidogyne incognita,* race 3. We utilized new genomic tools and resources developed for sweetpotato genetic improvement, which include chromosome-scale reference genome assembly of a putative diploid ancestral progenitor of sweetpotato, *I. trifida* (Wu et al. 2018); a quantitative reduced representation sequencing-based genotyping platform, GBSpoly (Wadl et al. 2018); and R packages for linkage map construction, MAPpoly (Mollinari and Garcia 2019; Mollinari et al. 2020), and QTL mapping, QTLpoly (Pereira et al. 2020).

The high-density integrated genetic linkage map was developed from a segregating mapping population consisting of 244 individuals derived from a cross between ‘Tanzania,’ an African landrace cultivar, and ‘Beauregard,’ a major US cultivar (Cervantes-Flores 2006). ‘Tanzania’ is highly resistant to four major RKN races, while ‘Beauregard’ is highly susceptible to RKN infection (Cervantes-Flores et al. 2002). Here, we describe the localization of a major QTL associated with resistance and their associated genetic effects. We further BLAST search the sequences within the confidence interval of the major QTL-associated markers in order to find putative candidate genes that may be involved in RKN resistance in sweetpotato.

## Materials and methods

### Germplasm

The TB mapping population was previously described in detail by Cervantes-Flores et al. (2008a). It consists of 244 individuals derived from a cross between the RKN resistant African cultivar, ‘Tanzania,’ and RKN susceptible USA cultivar, ‘Beauregard.’ ‘Tanzania’ is a released landrace sweetpotato cultivar in Uganda and is an important cultivar in sub-Saharan Africa. It is a cream-fleshed, high dry matter (ca. 30%) sweetpotato. ‘Beauregard’ is a major sweetpotato in the U.S and is an orange-fleshed, low dry matter (ca. 18%) cultivar. To develop the mapping population, crosses were made by hand in the screenhouse using ‘Tanzania’ as the female parent and ‘Beauregard’ as the male. Since ‘Tanzania’ has low pollen production, reciprocal crosses were not done. Out of the 350 seed that were germinated from this cross, a total of 250 clones (including 2 parents and 4 checks) were selected randomly for genetic studies. Since its development, the mapping population and parents have been maintained in tissue culture for long term storage, and in the greenhouse in a vegetative state in virus-free conditions. We conduct periodic propagation renewals with each clone planted, in a 20.3-cm-diameter pot containing Fafard P4 soil mix (Fafard, Agawam, MA). For each individual clone, five three-node cuttings were taken and planted into 72-cell Landmark™ seedling trays (Stuewe & Sons, Corvallis, OR) containing Fafard P4 for propagation in the greenhouse. Along with the two parents, we included four checks of known RKN performance (‘Covington’—resistant, ‘Hernandez’—partially resistant, ‘Jewel’—resistant and ‘Porto Rico’—susceptible). Plants were grown under greenhouse conditions at 25–28 °C and watered as needed. They were also fertilized to supplement their nutrient needs throughout the growing period.

## RKN screening in the greenhouse

A single ~ 15-cm-long cutting of each genotype was transplanted into 4′′ Azalea pots (round) containing a 50:50 pasteurized mix (by volume) of coarse sand and field soil (88.9% loamy sand, 8.3% silt and 2.8% clay), respectively (Cervantes-Flores et al. 2008a, 2002). The cuttings were allowed to root for 14 days before inoculation. The experiment was performed using a completely randomized design (CRD) with five replications (reps) separated by time (i.e., one replication consisted of all 244 full sibs plus two parents and four checks with reps repeated over time). All reps were planted and harvested in Fall 2016 on separate dates, with reps 1 through 5 harvested 62, 55, 62, 69 and 62 days after planting, respectively.

Root-knot nematodes eggs, *M. incognita* (race 3, the most predominant in North Carolina), that were previously cultured on ‘Rutgers’ tomato plants (*Solanum lycopersicum* L.) were extracted using NaOCl. This method has been described by Hussey and Barker (1973) and was used in this same population by Cervantes-Flores et al. (2006). A 15 ml inoculum solution containing ~ 20,000 RKN eggs was applied into the soil mixture to infest each plant. Plants were grown under controlled greenhouse conditions of 25–28 °C and were watered and fertilized as needed.

At the end of each trial (rep), the plants were harvested and rated by counting the number of root knots (galls) present on each root system for every genotype (Cervantes-Flores et al. 2002). This was done beginning with the first rep, then after one week the second, then the third and so on. The root tissue was stained with red food coloring (McCormick and Co., Baltimore, MD) to facilitate visual ratings. This method is non-toxic, and its results are comparable to those described by Hussey and Barker where a neurotoxic chemical, Phloxine B, was used (1973).

We also collected data for vine and root weights over four replications (reps 2 through 5). At the end of the greenhouse trial and the RKN assay, individual clones were placed in labeled paper bags and dried in a hot air oven at 65–70 °C for 72 h. Measurements were taken for the weights (in grams) of fresh and dry vine and roots (both storage and adventitious roots). This was done to determine whether there was a relationship between vine/root weight and number of RKN galls.

## Genotyping and genetic map construction

Genotyping and genetic map construction was performed as described by Amankwaah (2019). In brief, genotyping of the TB population was done using GBSpoly, an optimized genotyping-by-sequencing method for highly heterozygous polyploid genomes (Wadl et al. 2018). Genomic DNA library preparation and sequencing were done by the Genomic Sciences Laboratory (GSL) at North Carolina State University. GBSpoly reads were aligned against the *I. trifida* genome (Wu et al. 2018), and read counts were stored in VCF files using Tassel4-Poly (Pereira et al. 2018). Marker dosage calling was performed using SuperMASSA software (Serang et al. 2012) through VCF2SM software (Pereira et al. 2018).

The R package MAPpoly (Mollinari and Garcia 2019; available at https://github.com/mmolina/mappoly) was used to construct an integrated, high-density linkage map consisting of 14,813 markers spanning 2120 cM (6.9 loci/cM) of 15 linkage groups (LGs). The integrated, fully phased TB linkage map is available at https://gt4sp-genetic-map.shinyapps.io/tb_map/. This method has been comprehensively described by Mollinari et al. (2020), whereas the TB map has been fully described by Amankwaah et al. (2019). The genotype conditional probabilities were calculated every 1 cM for the whole genome (Mollinari et al. 2020) for QTL mapping purposes.

## Phenotypic data and QTL analysis

Restricted maximum likelihood (REML) variance component analysis of the phenotypic data was performed using ASReml-R version 4.1 (VSN International 2014) to generate joint adjusted means for QTL analysis using the following mixed model:

$$y_{ij} = \mu + r_{j} + t_{i} + e_{ij}$$

where $y_{ij}$ is the observed phenotypic value of genotype $i$ in rep $j$, $\mu$ is the population mean of the trait, $r_{j}$ is the random effect of replication $j$ $(j = 1, \ldots ,J;J = 4 \;{\text{or}}\; 5)$ depending on the trait) with $r_{j} \;\sim\;N\left( {0, \sigma_{r}^{2} } \right)$, $t_{i}$ is the fixed effect of individual $i$ $(i = 1, \ldots , I;I = 250)$ and ${e}_{ij}$ is the residual error with $e_{ij} \;\sim\;N\left( {0, \sigma_{e}^{2} } \right)$.

For heritability estimation, the effect of individuals $(t_{i} )$ was separated into two groups: random genotype effect $g_{i} \;\sim\;N\left( {0,\sigma_{g}^{2} } \right)$ $(i = 1, \ldots ,I_{g} ;\;I_{g} = 244)$ and fixed check effect $c_{i}$ $(i = I_{g} + 1, \ldots ,I_{g} + I_{c} ;\;I_{c} = 6)$. The mean-based broad-sense heritability values of root-knot (galls) counts, and vine and root weights in the greenhouse trial were estimated using the formula:

$$H^{2} = \frac{{\sigma_{g}^{2} }}{{\sigma_{g}^{2} + \frac{{\sigma_{e}^{2} }}{J}}}$$

For QTL mapping, we used a random-effect multiple interval mapping (REMIM) model implemented in the R package QTLpoly (Pereira et al. 2020; available at https://github.com/guilherme-pereira/qtlpoly). The variance components associated with the putative QTL were estimated using the REML method and tested using score statistics. Briefly, QTLpoly is based on the following stepwise method: First, one QTL at a time was added to the QTL model using forward search and a less conservative genome-wide significance level $(\alpha = 0.20)$. Second, a test of each added QTL conditional to all the others in the model was carried out using backward elimination with a more conservative genome-wide significance level $(\alpha = 0.05)$. These forward and backward procedures were repeated under the more conservative threshold until there were no more QTL added or dropped from the model. A region of 15 cM on either side of QTL already in the model was avoided in the search for new QTL. Finally, estimates of QTL allelic effects were computed based on the prediction of individual breeding values. Genome-wide significance levels $(\alpha )$ were defined based on a score-based resampling method (Pereira et al. 2020).

QTL peaks were identified from the most significant *P* values and plotted along the LGs. A final analysis included the computation of QTL heritability $\left( {h_{{{\text{QTL}}}}^{2} } \right)$ as a proportion of the genetic variance due to a QTL over the total phenotypic variance. The additive allele effects (contribution of each haplotype to the population mean) were obtained by averaging the genotypic values containing them. Since an identified QTL could possibly span a large portion of a LG, QTLpoly plots 95% confidence intervals for each identified QTL. These support intervals show the regions on the linkage group within which there is high confidence for the presence of the QTL.

## Candidate gene search

We searched for genes within support intervals of major QTL using sequence information from the pseudomolecules of the *I. trifida* (NSP306) reference genome (Wu et al. 2018). The *I. trifida* assembly is a 462 Mb reference sequence for hexaploid sweetpotato, with 32,301 annotated high confidence gene models (http://sweetpotato.plantbiology.msu.edu/). This reference sequence genome was repeat masked then annotated for protein-coding genes using a set of transcript and protein evidence (Wu et al. 2018).

## Results

### Phenotypic data

**Fig.1:**
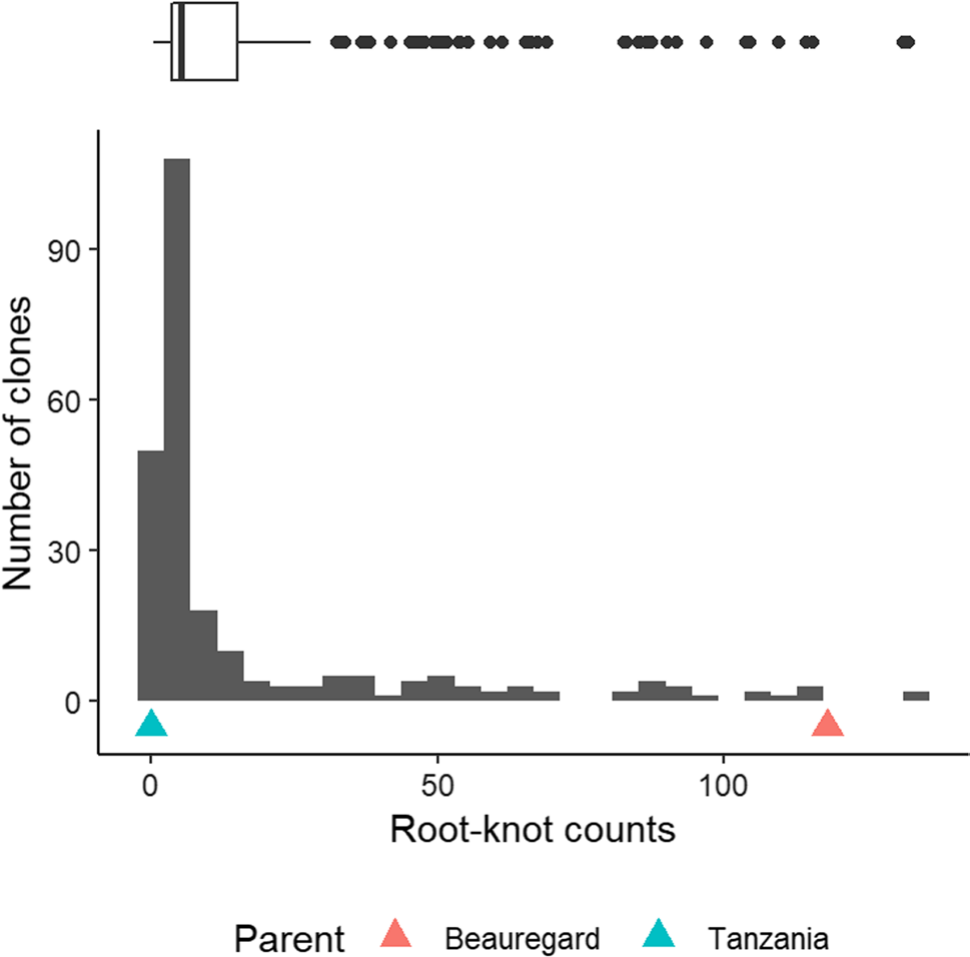
Histogram and boxplot of the adjusted means of root-knot counts in the TB population. Most of the progeny (75%) reacted like ‘Tanzania’ (< 15 root-knot counts), the resistant parent, while < 1% were more susceptible than ‘Beauregard.’ The boxplot above the histogram shows the statistics of the distribution

A histogram plot of the adjusted mean of the root-knot count data from five replications showed that most of the progenies were resistant (Fig. 1). The correlation among different replications and the adjusted mean were high ($r$= 0.79–0.96; *P* < 0.001), and there was no significant difference among reps despite their distribution over time (Supplemental Figure S1). Just like in individual reps, the distribution of joint means over all five trials demonstrated skewing toward the resistant parent (Fig. 1). Transgressive segregation was observed, i.e., a few progenies (< 1%) exhibiting lower levels of RKN resistance than ‘Beauregard,’ the susceptible parent (more than 118 galls per root system). The resistant parent, ‘Tanzania’ demonstrated 100% resistance in that there were no root-knot galls observed in its entire root system. Mean-based broad-sense heritability in the greenhouse trial was estimated at 96% for root-knot counts.

Histograms of the adjusted means for fresh and dry vine and root weights in the parents and progeny of the TB population (Supplemental Figure S2) showed that most genotypes segregated toward the female parent, ‘Tanzania.’ Dry vine weight was normally distributed, whereas dry root weight was right skewed. The weight of vines for ‘Tanzania’ was high, but it did not have any storage roots formed by the end of the greenhouse trial, just like most progenies. We did not observe any significant correlation (*P* value > 0.05) between root-knot counts and any other measured trait (Supplemental Figure S3). Heritability values ranged from 55 to 68% for dry weight of vines and storage roots, respectively. Raw phenotypic data and the adjusted means are available in Supplemental File S1.

## QTL analysis

**Fig. 2:**
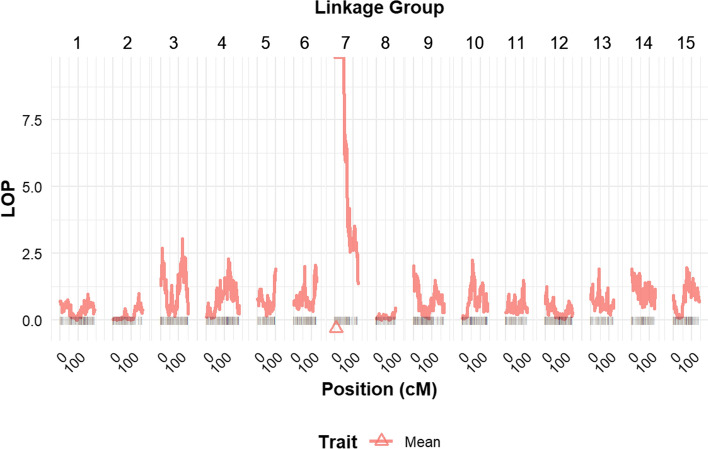
QTL profile for root-knot count adjusted mean in the TB population. A single major QTL was identified on linkage group 7. LOP is the logarithm of *P* values of score tests carried out every centimorgan (cM)

By using score-based statistics and a random-effect QTL model implemented in the R package QTLpoly, we detected a single major QTL (*P* value < 2.22e−16) for resistance to RKN, *M. incognita* race 3, on LG 7 at 6.74 cM (Fig. 2), explaining 58.3% of the total phenotypic variation (Table 1). Similarly, this QTL was consistently present in each of the reps (Supplemental Figure S4). The support intervals for the mapped QTL (Table 1; Supplemental Figure S5) ranged from 0 to 49.01 cM on the LG 7. We did not observe any significant QTL with the vine or storage roots weight traits (data not shown). Since the plants were put in 4′′ round Azalea pots for the greenhouse assay and harvested between 55 and 69 days after planting, we believe that these weight traits were measured at an early stage before the plants had enough time to differentially express themselves fully and more consistently for genetic differences to be realized.

**Table 1:** Summary statistics of QTL for RKN resistance on the TB map. ${h}_{\mathrm{QTL}}^{2}$ is the heritability of the QTL

	LG	Position (cM)	Marker (bp)	Score	*P* value	Mean ($\mu$)	${h}_{\mathrm{QTL}}^{2}$
QTL	7	6.74	S7_976713 (976713)	691.86	< 2.22e–16	15.77	0.583
Lower SI	7	0.00	S7_55468 (55468)	596.41	< 2.22e–16		
Upper SI	7	49.01	S7_4668738 (4668738)	339.39	< 2.22e–16		

Lower and upper support intervals (SI) represent the boundaries upstream and downstream of the QTL on the linkage group (LG). Score represents the score statistics for the marker

**Fig. 3:**
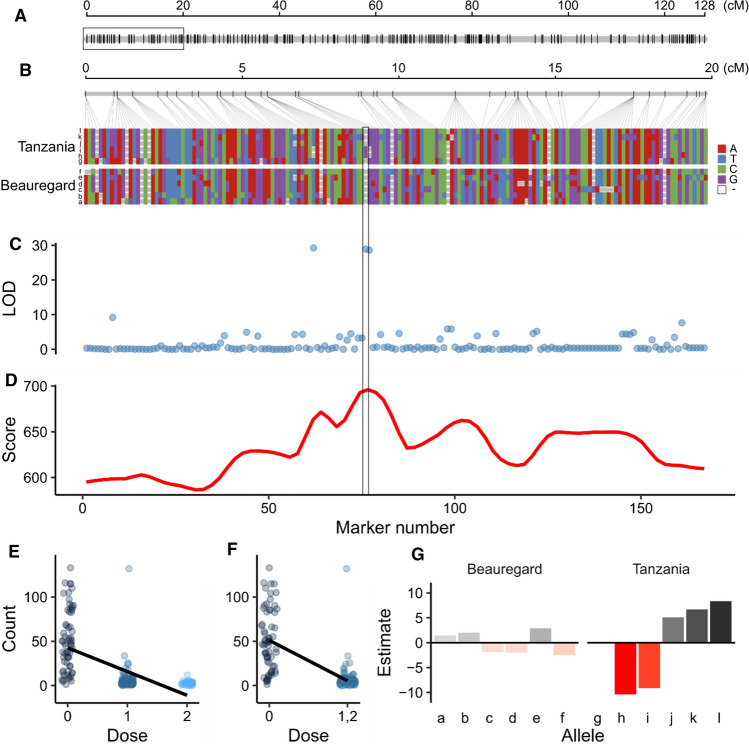
Root-knot nematode (RKN) resistance locus in TB mapping population. **a** Linkage group (LG) 7. **b** The first 167 markers (1–20 cM of LG 7) show together with their allele variants, dosage and linkage phasing. From bottom to top, *a* through *f* and *g* through *l* represent ‘Beauregard’ and ‘Tanzania’ haplotypes, respectively. **c** Single-marker analysis has shown three highly significant markers (LOD ≈ 30), whereas **d** random-effect interval mapping has shown that all markers are highly significant (score test > 550, *P* value < 2.2e–16). **e** Regression on the marker dosage and **f** on the diploidized genotype call for S7_1038803. **g** Breakdown of genotypic values showing additive effects (as deviation from population mean, $\mu$ = 15.8) from each allele at the major QTL position (S7_1038803). Note how duplex markers in ‘Tanzania’ are in coupling-phase with *h* and *i* haplotypes at the highlighted locus in Fig. 3b

For the major QTL region on LG 7 (Fig. 3a), we conducted single-marker analysis incorporating marker dosage using random-effect interval mapping (score tests on the genotype conditional probability) on each of the 167 markers located within the first 20 cM (Fig. 3b). Three markers (S7_899372, S7_1038803 and S7_1038845) exhibited LOD score ca. 30, representing the highest among the 167 single markers analyzed (Fig. 3c). But, score tests showed that all markers in the QTL interval were significant (*P* value < 2.22e–16), with the highest score associated with marker S7_1038803 (Fig. 3d).

Interestingly, the three most significant markers from single-marker analysis were double-dose (duplex) for ‘Tanzania,’ and zero-dose (nulliplex) for ‘Beauregard’ relative to an arbitrary allele *A*. Such a cross, *AAaaaa* × *aaaaaa*, is expected to segregate 1 *aaaaaa*: 3 *Aaaaaa*: 1 *AAaaaa*. For instance, for marker S7_1038803, in which the observed segregation ratio was 64:114:39, there was not statistically significant deviation from the expected 1:3:1 segregation ratio (${\chi }^{2}$ = 12.24, *P* value > 0.001). For this marker, the linear regression on the three dosage classes (0, 1, 2) is shown in Fig. 3e with a highly significant slope (${R}^{2}$ = 0.383; *F*-statistic = 133.1, *P* value < 2.22e–16). There was no observed significant difference between the heterozygous class means (*F*-statistic = 2.7, *P*-value = 0.101), and combining these two classes (1, 2) as in Fig. 3f also resulted in a significant slope (${R}^{2}$ = 0.500; *F*-statistic = 214.3, *P* value < 2.22e–16). Finally, we fitted a Haley–Knott regression (Haley and Knott 1992) using the map-based genotypic probabilities on S7_1038803 marker, with an interaction between alleles *h* and *i* (i.e., dominance effect), and this interaction appeared to be highly significant ($P$ value < 5.5e–14) (Supplemental Table S1).

From the breakdown of genotypic values derived from the random-effect interval mapping showing additive effects of each parental haplotype (Fig. 3g), we noticed that the segregating alleles for root-knot counts were mostly present in ‘Tanzania,’ the resistant parent, whereas ‘Beauregard’ did not seem to contribute much to the trait variation. Alleles with positive effects demonstrated how much susceptibility to RKN can be increased by the presence of those alleles in the population, whereas those with a negative effect show how much resistance to RKN can be increased. We were able to identify two haplotypes in ‘Tanzania,’ *h* and *i*, that were the most important configurations in increasing resistance. In fact, the significant duplex markers in ‘Tanzania’ identified as highly significant by single-marker analysis were in coupling-phase with *h* and *i* haplotypes.

## Candidate gene search

The QTL for RKN resistance on LG 7 spanned a wide range of the chromosome that included a number of genes and several other molecular components that regulate them. Our search was performed on LG 7 within the ~ 50 cM support interval of the mapped QTL peak and spanned 4.61 Mb (~ 20%) of chromosome 7 of *I. trifida* reference genome (Wu et al. 2018) which contained 629 annotated genes (Supplemental File S2). The first half of the LG 7 in TB was highly colinear with the chromosome 7 of *I. trifida* (Mollinari et al. 2020), which allowed us to use the same annotation for *I. batatas*. As there were too many genes to manually curate and 50 cM being a big genomic space, we decided to present all of these 629 annotated genes as supplementary material (Supplemental File S2) that should be probed further in order to identify those that are most relevant to RKN resistance. However, genes that were identified in the region of the QTL peak from 5 to 10 cM (i.e. from S7_811167 to S7_1565091) and may be related to nematode resistance included: a major facilitator protein (itf07g03290.t1; gene name assigned on the *I. trifida* reference genome), a superfamily of membrane transport proteins that facilitate movement of sugars and small molecules across cell membranes (Pao et al. 1998); vacuolar protein sorting (itf07g04740.t2), a family of proteins involved in a series of vital mechanisms including, but not limited to autophagy, ion secretion by salt glands, abiotic/biotic stress responses, and so on (Surpin and Raikhel 2004; Xiang et al. 2013); phosphatidylinositol transfer protein (itf07g06330.t1), a family of proteins responsible for various cellular functions including development and signaling, involvement in exocytosis, reactive oxygen species (ROS) production, vesicular traffic and transcriptional activity (Park et al. 2003; Joo et al. 2005; Lee et al. 2008); and a Scarecrow-like transcription factor (itf07g01620), which has been reported by Huang et al. (2005) as a putative target for a bioactive nematode peptide in Arabidopsis.

## Discussion

The TB mapping population has been studied extensively and is well suited for investigating the architecture of resistance to RKN for various reasons, as discussed by Cervantes-Flores et al. (2002, 2005, 2008b). In all screening studies of several sweetpotato genotypes, ‘Tanzania’ has consistently shown high levels of resistance to multiple species and races of nematodes (Cervantes-Flores et al. 2002; Karuri et al. 2017). ‘Beauregard,’ on the other hand, is susceptible to the same RKN species (Cervantes-Flores et al. 2002). The F_1_ mapping population is large enough at 244 individuals to provide enough statistical power of detection and estimation of QTL effects, and also allows for detection of recombinant genotypes that allow better dissection of the trait and QTL analysis. The importance of population size to study the inheritance of a trait and to detect QTL has been strongly emphasized by several researchers (Collard et al. 2005; Doerge et al. 1997; Cervantes-Flores et al. 2008a). In a polyploid crop such as sweetpotato, a large population size increases the statistical power and resolution to detect QTL (Collard et al. 2005; Kriegner et al. 2003; Cervantes-Flores et al. 2008b). The previous studies have relied on marker data that are not fully informative since the marker assays lacked the ability to capture the full spectrum of allele dosage. For a complex polyploid like sweetpotato, it is important to utilize higher-dose markers because these markers can help to link different homeologous chromosomes and construct an integrated map (Garcia et al. 2013; Mollinari and Garcia 2019). Additionally, in order to guarantee reasonable coverage of the relatively large genome of hexaploid sweetpotato for genetic characterization and analysis, a relatively large number of markers (several thousands) are required to capture the full spectrum of recombination events.

The frequency distribution observed in our study for root-knot counts (Fig. 1) was consistent with that of other studies (Cervantes-Flores et al. 2008b; Nakayama et al. 2012) and, given the percentage of resistant (~ 75%) progenies, conforms to 4:1 ratio. This pattern can be explained by the action of one or two major genes/alleles that are most likely segregating independently (Cervantes-Flores et al. 2008b), assuming hexasomic inheritance. Proposing the hypothesis of a single double-dose QTL, it is expected that 80% of the progeny contain at least one resistant allele (60% one allele, 20% two allele, 20% zero allele). This is in strong agreement with our observation of the phenotype (Fig. 1). However, since a continuum of resistance reactions is observed in most of the segregating populations studied to date, the resistance reaction to RKN is considered quantitative, but most likely conferred by additional loci with small effects. Polygenic resistance to nematodes is very common in plants and has been reported in sweetpotato (Cervantes-Flores et al. 2008b) and potato [*Solanum tuberosum*] (Bryan et al. 2002).

Molecular markers linked to a QTL that have been effectively utilized in breeding programs for cultivar development include QTL for soybean resistance to cyst nematode [*Heterodera glycines*] (Concibido et al. 2004), the *Fhb1* QTL for Fusarium head blight resistance in wheat [*Triticum aestivum*] (Anderson et al. 2008), the *H1* QTL for potato cyst nematode resistance in potato [*Solanum tuberosum* spp.] (Finkers-Tomczak et al. 2011), the *Mi* QTL for root-knot nematode resistance in tomato [*Solanum lycopersicum*] (Ho et al. 1992) and the *Sub1* QTL for submergence tolerance in rice [*Oryza sativa*] (Septiningsih et al. 2009). In each of these cases, the favorable QTL allele had an effect that was large enough to be easily tracked and fixed by standard breeding procedures (Bernardo 2016).

The broad-sense heritability of resistance to RKN was high (${H}^{2}$ = 96%) and this is close to the 89% observed by Cervantes-Flores et al. (2008b) considering that a greater portion of the progeny would be considered resistant according to the number of root-knot galls present in the root system. Here, we identified one major resistance QTL on LG 7 (Fig. 2) explaining 58.3% of the variation for root-knot counts in the mapping population (Table 1). Furthermore, the QTL described for the joint adjusted means were consistent with those of the raw count data of individual replications (Supplemental Figure S4).

In their earlier work on RKN resistance in this TB population, Cervantes-Flores et al. (2008b) used AFLP markers and identified nine minor effect QTL responsible for the resistance. They reported that none of the mapped QTL explained more than 15% of variation, but interestingly observed three unmapped duplex markers that explained ~ 45% of the variation. These duplex markers might be in coupling-phase with the three duplex markers identified via single-marker analysis here (Fig. 3C). With this information, they concluded that these higher-dose markers are potentially associated with one or two major genes and that this would agree with the observed segregation ratio of the phenotypes. Although they could not determine this hypothesis at that point in time, they recommended further analysis through the addition of more markers, sequencing and other such approaches (Cervantes-Flores et al. 2008b).

The QTL on LG 7 explaining 58.3% of the phenotypic variation is a great improvement compared to the work of Cervantes-Flores et al. (2008b), where none of their QTL identified in the linkage map explained more than 15% of the phenotypic variation. This was only possible because, in contrast to the map by Cervantes-Flores et al. (2008a), our integrated TB genetic map was developed using single- and multi-dose SNP and indel markers (Amankwaah 2019). The fully phased linkage map showed that ‘Tanzania’ was the greatest contributor providing a combination of favorable alleles that increased resistance (Fig. 3g). The existence of two haplotypes contributing to resistance explains the fact that more than one QTL was marginally mapped by Cervantes-Flores et al. (2008b), when, in fact, it consisted of a single locus. In addition, either simplex or duplex individuals showed similar levels of resistance (Fig. 3f). Thus, we estimated the dominance effect of alleles *h* and *i* using a Haley–Knott regression (Haley and Knott 1992) with interaction between them. The results showed a highly significant interaction, with allelic substitution effect of *h* and *i* equals –49.0 and –49.3, respectively, and interaction effect equals 50.0 indicating that the presence of either allele decrease ~ 49 the number of root-knot count; however, in the presence of both alleles this number is practically unaffected (Supplemental Table S1). These findings indicate that such an RKN resistance locus is most likely complete dominant in nature.

The mapped QTL locations on the linkage groups were considered as potential regions to search for putative candidate genes that might be involved in RKN resistance, with QTLpoly providing the confidence limits for the QTL (Table 1; Supplemental Figure S5) within which a search for candidate genes could be conducted. A strong marker–QTL association detected in full-sib progenies could be an important factor for crop improvement programs that use clonal propagation because the possibility of crossover between the marker and QTL is low. Sweetpotato has high genetic complexity and its genome is not yet fully sequenced to date; therefore, inferences about putative candidate genes could contribute to new insights and open new areas of research in mining and validation of QTL and genes of interest (Balsalobre et al. 2017). Our study, as well as that of Gemenet et al. (2020) who studied the molecular relationship between β-carotene and starch content in sweetpotato, validates the robustness of the new genomics tools in dissecting the genetic architecture of important yet complex traits in cultivated sweetpotato.

Upon inspection of the *I. trifida* reference genomic region corresponding to our QTL, we observed that several genes were centered within the major QTL peak. We recommend that this sequence region be probed further and analyzed using RNA-seq data or other fine mapping and gene cloning approaches to identify the exact genes of importance in identifying resistance genes to RKNs. Resistance of field plants against RKN has previously been observed to be mediated through a hypersensitive response in cowpea (Das et al. 2008), tomato (Teresa Melillo et al. 2006) and sweetpotato (Dean and Struble 1953; Gentile et al. 1962; Martin and Birchfield 1973; Jones and Dukes 1980).

Favorable alleles have an average effect that decrease the mean of the trait value, thus increasing resistance. ‘Tanzania,’ being the resistant parent, was shown to have alleles that are able to mask its unfavorable alleles, thereby resulting in resistance. When making selection in a breeding program, the use of an identified QTL requires the integration of breeding values estimated from average effects of alleles. Selection is therefore made on favorable alleles only whose additive effects can pass on to the next generation for population improvement.

Based on our analyses of the breeding value predictions in the TB population using QTLpoly, the top ten genotypes (i.e., clones) that have a favorable allele effect for decreasing the mean of RKN egg masses and thus increasing resistance were: TB163, TB237, TB83, TB63, TB183, TB50, TB214, TB213, TB249 and TB235 (Supplemental File S3). Based on these predictions, if these clones are used as parents for RKN population improvement, the average effects of their alleles would potentially increase the genetic gain for RKN resistance.

In conclusion, we have identified a major QTL associated with RKN resistance in hexaploid sweetpotato that explain 58.3% of the variation in gall production. Our understanding of the genetic architecture of this important trait has significantly improved due to the utilization of new molecular and bioinformatics tools. The search of possible candidate genes for mapped QTL is a preliminary analysis highlighting the importance of new insights into the relationships between statistical genetics and biology. Further research is needed to expound on the observations made in this study, to identify and confirm the genes at these QTL regions through RNA-seq analysis and transgenic approaches such as gene silencing and CRISPR technologies. The work presented here is one aspect of a larger effort focused on the development and efficient use of new genomic, statistical and bioinformatics tools for sweetpotato improvement under the GT4SP project.

## Supplementary Information

Supplementary file1 (CSV 51kb)

Supplementary file1 (XLSX 108kb)

Supplementary file1 (CSV 4kb)

Supplementary file1 (DOCX 235kb)

## References

[CR1] Agrios GN (2004). Plant diseases caused by nematodes. Plant pathology (fifth edition).

[CR2] Amankwaah VA 2019. phenotyping and genetic studies of storage root chemistry traits in sweetpotato. Raleigh (NC): North Carolina State University. PhD Dissertation.

[CR3] Anderson JA, Chao S, Liu S (2008). Molecular breeding using a major QTL for Fusarium head blight resistance in wheat. Crop Sci.

[CR4] Balsalobre TWA (2017). GBS-based single dosage markers for linkage and QTL mapping allow gene mining for yield-related traits in sugarcane. BMC Genomics.

[CR5] Barr AR, Chalmers KJ, Karakousis A, Kretschmer JM, Manning S, Lance RCM (1998). RFLP mapping of a new cereal cyst nematode resistance locus in barley. Plant Breed.

[CR6] Bernardo R (2016). Bandwagons I, too, have known. Theor Appl Genet.

[CR7] Bryan G (2002). Mapping QTLs for resistance to cyst nematode *Globodera pallida* derived from the wild potato species Solanum vernei. Theor Appl Genet.

[CR82] Cervantes-Flores JC (2000) Root-knot nematode resistance in sweetpotato and development of sweetpotato differential host genotypes for *Meloidogyne* spp. Raleigh (NC): North Carolina State University. PhD Dissertation

[CR8] Cervantes-Flores JC (2006) Development of a genetic linkage map and QTL analysis in sweetpotato. Raleigh(NC): North Carolina State University. PhD Dissertation

[CR9] Cervantes-Flores JC, Yencho GC, Davis EL (2002). Host reactions of sweetpotato genotypes to root-knot nematodes and variation in virulence of *Meloidogyne incognita* populations. HortScience.

[CR10] Cervantes-Flores JC (2008). Development of a genetic map and identification of homologous linkage groups in sweetpotato using multi-dose AFLP markers. Mol Breed.

[CR11] Cervantes-Flores JC, Yencho GC, Pecota KV, Sosinski B (2008). Detection of quantitative trait loci and inheritance of root-knot nematode resistance in sweetpotato. J Am Soc Hortic Sci.

[CR12] Collard BCY, Jahufer ZZ, Brouwer JB, Pang ECK (2005). An introduction to markers, quantitative trait loci (QTL) mapping and marker-assisted selection for crop improvement: The basic concepts. Euphytica.

[CR13] Concibido VC, Diersb BW, Arelli PR (2004). A decade of QTL mapping for cyst nematode resistance in soybean. Crop Sci.

[CR14] Cordner HB, Struble FB, Morrison L (1954). Breeding sweetpotatoes for resistance to the root-knot nematode. Plant Dis Rptr Suppl.

[CR15] Coyne DL, Kagoda F, Wambugu E (2006). Response of cassava to nematicide application and plant-parasitic nematode infection in East Africa, with emphasis on root knot nematodes. Int J Pest Manag.

[CR57] Das S, DeMason DA, Ehlers JD, Close TJ, Roberts PA (2008). Histological characterization of root-knot nematode resistance in cowpea and its relation to reactive oxygen species modulation. J Exp Bot.

[CR61] Dean JL, Struble FB (1953). Resistance and susceptibility to rootknot nematodes in tomato and sweet potato. Phytopathology.

[CR16] Doerge RW, Zeng ZB, Weir BS (1997). Statistical issues in the search for genes affecting quantitative traits in experimental populations. Stat Sci.

[CR59] Finkers-Tomczak A, Bakker E, de Boer J, van der Vossen E, Achenbach U, Golas T, Suryaningrat S, Smant G, Bakker J, Goverse A (2011). Comparative sequence analysis of the potato cyst nematode resistance locus H1 reveals a major lack of co-linearity between three haplotypes in potato (Solanum tuberosum ssp.).. Theor Appl Genet.

[CR17] Gemenet DC (2020). Quantitative trait loci and differential gene expression analyses reveal the genetic basis for negatively associated β-carotene and starch content in hexaploid sweetpotato [*Ipomoea batatas* (L.) Lam.]. Theor Appl Genet.

[CR18] Dropkin VH (1969). Cellular responses of plants to nematode infections. Annu Rev Phytopathol.

[CR19] Garcia AAF (2013). SNP Genotyping allows an in-depth characterization of the genome of sugarcane and other complex autopolyploids. Sci Reports.

[CR20] Gasapin RM (1984). Resistance of fifty two sweetpotato [Ipomoea batatas (L.) Lam] cultivars to *Meloidogyne incognita* and *M. javanica*. Annu Trop Res.

[CR62] Gentile AG, Kimble KA, Hanna GC (1962). Reactions of sweetpotato breeding lines to Meloidogyne species when inoculated by animproved method. Phytopathology.

[CR21] Giamalva M, Hernandez T, Martin W, Mill J (1961) Testing sweetpotato progenies for nematode resistance.In: Proc. Ann. Conv. Assn. Southern. Agr. Workers, 58(174)

[CR22] Haley CS, Knott SA (1992). A simple regression method for mapping quantitative trait loci in line crosses using flanking markers. Heredity.

[CR58] Ho JY, Weide R, Ma HM, van Wordragen MF, Lambert KN, Koornneef M, Zabel P, Williamson VM (1992). The root-knot nematode resistance gene (Mi) in tomato: construction of a molecular linkage map and identification of dominant cDNA markers in resistant genotypes. Plant J.

[CR23] Hussey R, Barker K (1973). A comparison of methods of collecting inocula of mwloidogyne spp., including a new technique. Plant Dis Rptr Suppl.

[CR24] Huang G, Dong R, Allen R, Davis EL, Thomas J, Hussey RS (2005). A root-knot nematode secretory peptide functions as a ligand for a plant transcription factor. Mol Plant-Microbe Interact.

[CR25] Jones A, Dukes PD (1980). Heritabilities of sweet potato resistance to root knot caused by *Meloidogyne incognita* and *Meloidogyne javanica*. J Am Soc Hort.

[CR81] Jatala P, Jansson RK, Raman KV (1991). Biology and management of plant-parasitic nematodes on sweet potato. Sweet potato pest management: a global perspective 1991..

[CR26] Joo JH (2005). Auxin-induced reactive oxygen species production requires the activation of phosphatidylinositol 3-kinase. FEBS Lett.

[CR27] Karuri HW (2017). A survey of root knot nematodes and resistance to *Meloidogyne incognita* in sweet potato varieties from Kenyan fields. Crop Prot.

[CR28] Komiyama A (2006). Resistance to two races of *Meloidogyne incognita* and resistance mechanism in diploid *Ipomoea trifida*. Breed Sci.

[CR29] Kriegner A (2003). A genetic linkage map of sweetpotato [*Ipomoea batatas* (L.) Lam] based on AFLP markers. Mol Breeding.

[CR30] Lawrence GW, Clark CA, Wright VL (1986). Influence of *Meloidogyne incognita* on resistant and susceptible sweetpotato cultivars. J Nematol.

[CR31] Lee Y (2008). Roles of Phosphatidylinositol 3-kinase in root hair growth. Plant Physiol.

[CR32] Makumbi-Kidza NN, Speijer PR, Sikora RA (2000). Effects of *Meloidogyne incognita* on growth and storage-root formation of cassava (Manihot esculenta). Suppl J Nematol.

[CR33] Martin WJ & Birchfield W (1973) Further observations of variability in *Meloidogyne incognita* on sweetpotato. Plant Dis Rep 57(199)

[CR34] Mcharo M (2005). Molecular marker variability for southern root-knot nematode resistance in sweetpotato. Euphytica.

[CR35] Mollinari M, Garcia AAF (2019). Linkage analysis and haplotype phasing in experimental autopolyploid populations with high ploidy level using hidden Markov models. Genes Genomes Genet.

[CR36] Mollinari M (2020). Unraveling the hexaploid sweetpotato inheritance using ultra-dense multilocus mapping. Genes Genomes Genet.

[CR37] Nakayama H (2012). Development of AFLP-derived SCAR markers associated with resistance to two races of southern root-knot nematode in sweetpotato. Euphytica.

[CR38] Namaganda JM, Gowen SR, Karamura EB (1993) Plant-parasitic nematodes associated with some root crops in Uganda. In: Proceedings of the first east and southern africa crop science conference. Kampala, African Crop Science Journal, pp. 312–314

[CR39] Okamoto K, Mitsui Y (1974). Occurance of resistance-breaking population of *Meloidogyne incognita* on tomato. Jpn J Nematol.

[CR83] Pao SS, Paulsen IT, Saier MH (1998). Major facilitator superfamily. Microbiol Mol Biol Rev.

[CR40] Park KY (2003). A role for phosphatidylinositol 3-phosphate in abscisic acid-induced reactive oxygen species generation in guard cells. Plant Physiol.

[CR41] Paulson RE, Webster JM (1972). Ultrastructure of hypersensitive reaction in roots of tomato, *Lycopersicon esculentum* L., to infection by root-knot nematode *Meloidogyne incognita*. Physiol Plant Pathol.

[CR42] Pereira GS, Garcia AAF, Margarido GR (2018). A fully automated pipeline for quantitative genotype calling from next generation sequencing data in autopolyploids. BMC Bioinform.

[CR43] Pereira GDS (2020). Multiple QTL mapping in autopolyploids: a random-effect model approach with application in a hexaploid sweetpotato full-sib population. Genetics.

[CR44] Sasser JN (1980). Root-knot nematodes: A global menace to crop production. Plant Dis.

[CR45] Sasser JN, Freckman DW, Veech JA, Dickson DW (1987). A world perspective on nematology: the role of the society. Vistas on nematology.

[CR46] Septiningsih EM (2009). Development of submergence-tolerant rice cultivars: the Sub1 locus and beyond. Ann Bot.

[CR47] Serang O, Mollinari M, Garcia AAF (2012). Efficient exact maximum a posteriori computation for bayesian SNP genotyping in polyploids. PLoS ONE.

[CR48] Surpin M, Raikhel N (2004). Traffic jams affect plant development and signal transduction. Nat Rev Mol Cell Biol.

[CR60] Teresa Melillo M, Leonetti P, Bongiovanni M, Castagnone-Sereno P, Bleve-Zacheo T (2006). Modulation of reactive oxygen species activities and H2O2 accumulation during compatible and incompatible tomato-root-knot nematode interactions. New Phytologist.

[CR49] Ukoskit K, Thompson P, Watson C, Lawrence G (1997). Identifying a randomly amplified polymorphic DNA (RAPD) marker linked to a gene for root-knot nematode resistance in sweetpotato. J Am Soc Hot Sci.

[CR50] VSN International (2014) GenStat for Windows 16th Edition

[CR51] Wadl PA, Olukolu BA, Branham SE, Jarret RL, Yencho GC, Jackson DM (2018). Genetic diversity and population structure of the USDA sweetpotato (Ipomoea batatas) germplasm collections using GBSpoly. Front Plant Sci.

[CR52] Wang D, Arelli PR, Shoemaker RC, Diers BW (2001). Loci underlying resistance to race 3 of soybean cyst nematode in Glycine soja plant introduction 468916. Theor Appl Genet.

[CR53] Whitehead AG (1998). Plant nematode control.

[CR54] Wu S (2018). Genome sequence of two diploid wild relatives of cultivated sweetpotato reveal targets for genetic improvement. Nat Commun.

[CR55] Xiang L, Etxeberria E, Van den Ende W (2013). Vacuolar protein sorting mechanisms in plants. FEBS J.

[CR56] Ynturi P (2006). Association of root-knot nematode resistance genes with simple sequence repeat markers on two chromosomes in cotton. Crop Sci.

