# Supplementary material for: Discovery of a major QTL for root-knot nematode (Meloidogyne incognita) resistance in cultivated sweetpotato (Ipomoea batatas)
Source: Theor Appl Genet. 2021 Apr 3;134(7):1945–55. doi: 10.1007/s00122-021-03797-z (PMC8263542; doi:10.1007/s00122-021-03797-z)
Supplement: Supplementary file 4 — Supplementary file1 (DOCX 235kb) [file 122_2021_3797_MOESM4_ESM.docx]

**
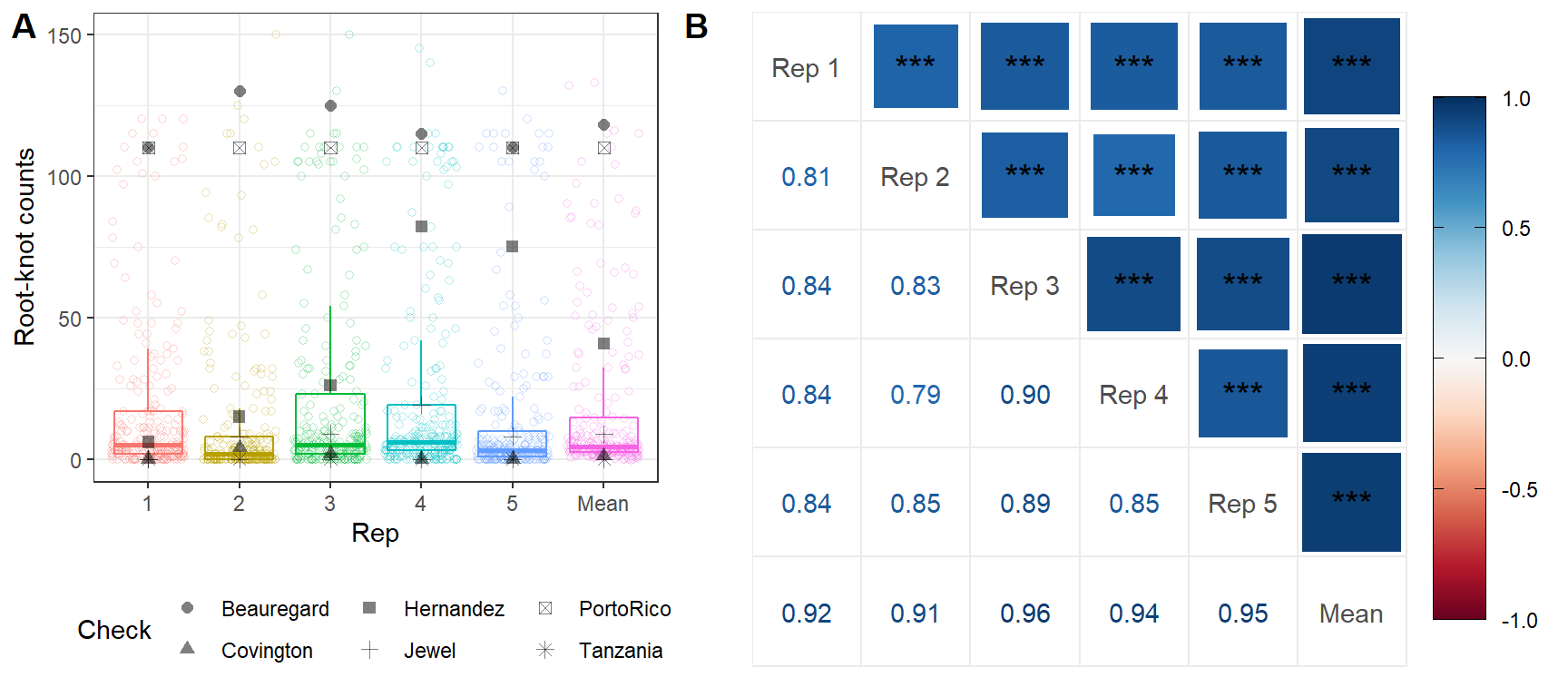
**

Supplemental Figure S1. Root-knot counts in the progeny of TB mapping population across different replications (reps 1 through 5) and the adjusted mean. (A) Boxplots showing no significant differences among reps. (B) Pearson correlation showing high correlation among reps and with the adjusted mean (****P* < 0.001).


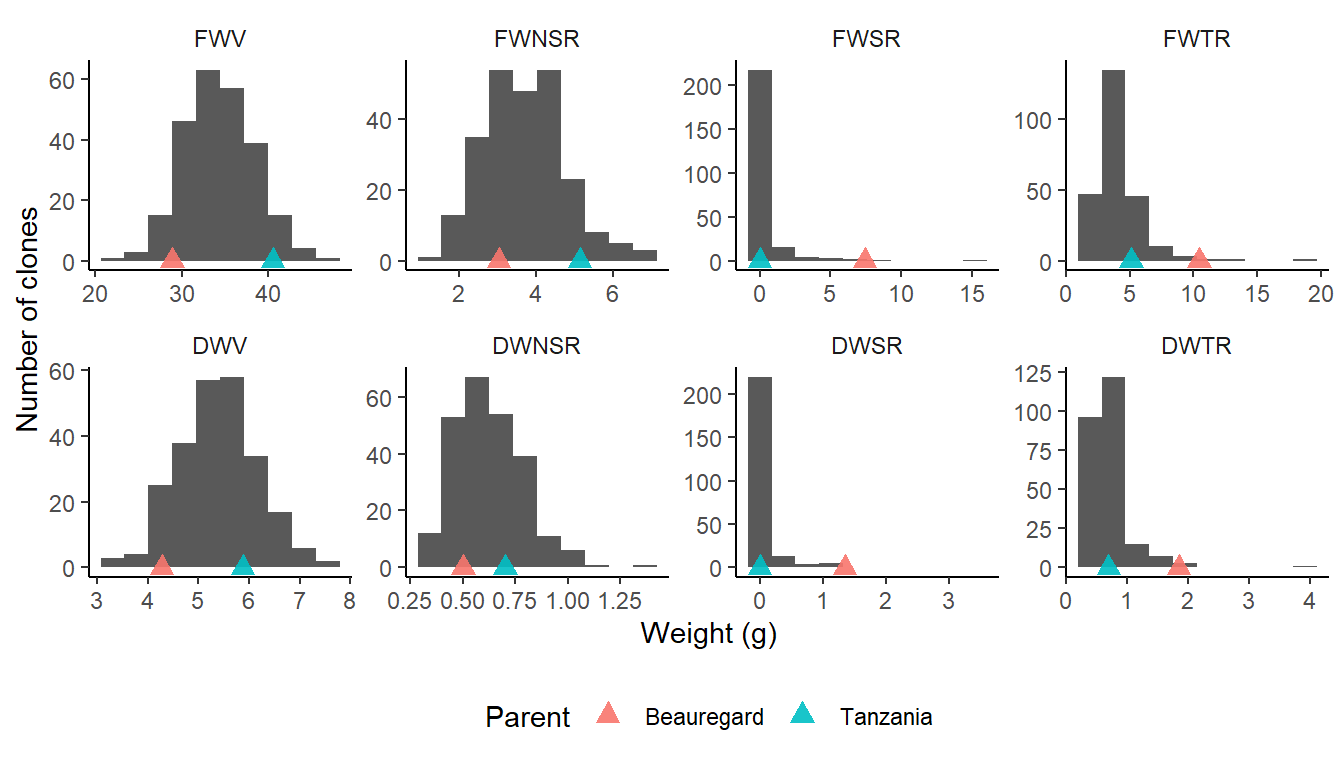
 Supplemental Figure S2. Histograms of adjusted means of fresh (FW) and dry (DW) weight of vine (V), non-storage root (NSR), storage root (SR) and total root (TR) in the parents and progeny of the TB population.


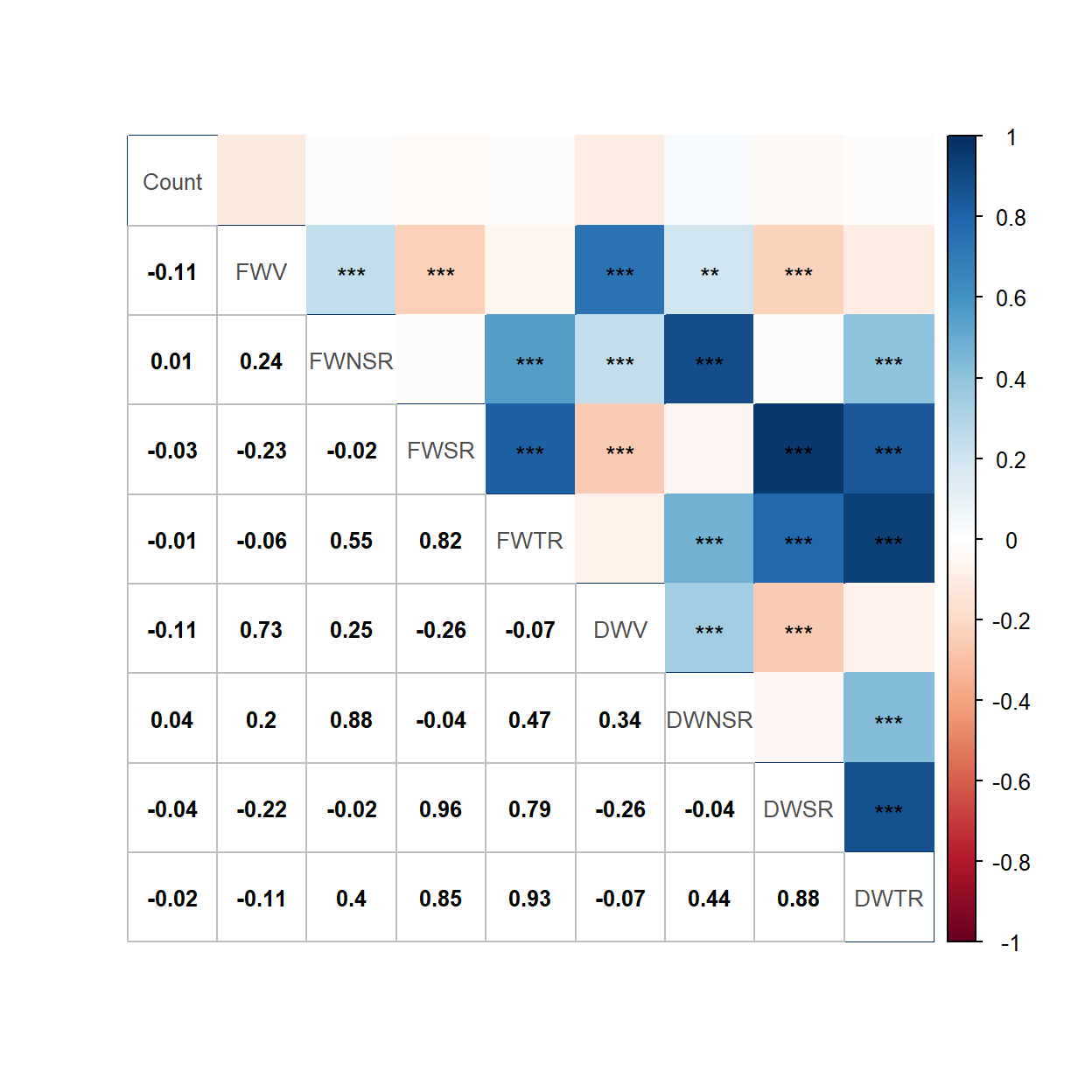


Supplemental Figure S3. Correlogram among adjusted means for root-knot counts (Count) and fresh (FW) and dry (DW) weight of vine (V), non-storage root (NSR), storage root (SR) and total root (TR) at 55~62 days after planting. Count is not significantly correlated to any of the weight traits (**P* < 0.05, ***P* < 0.01, ****P* < 0.001).


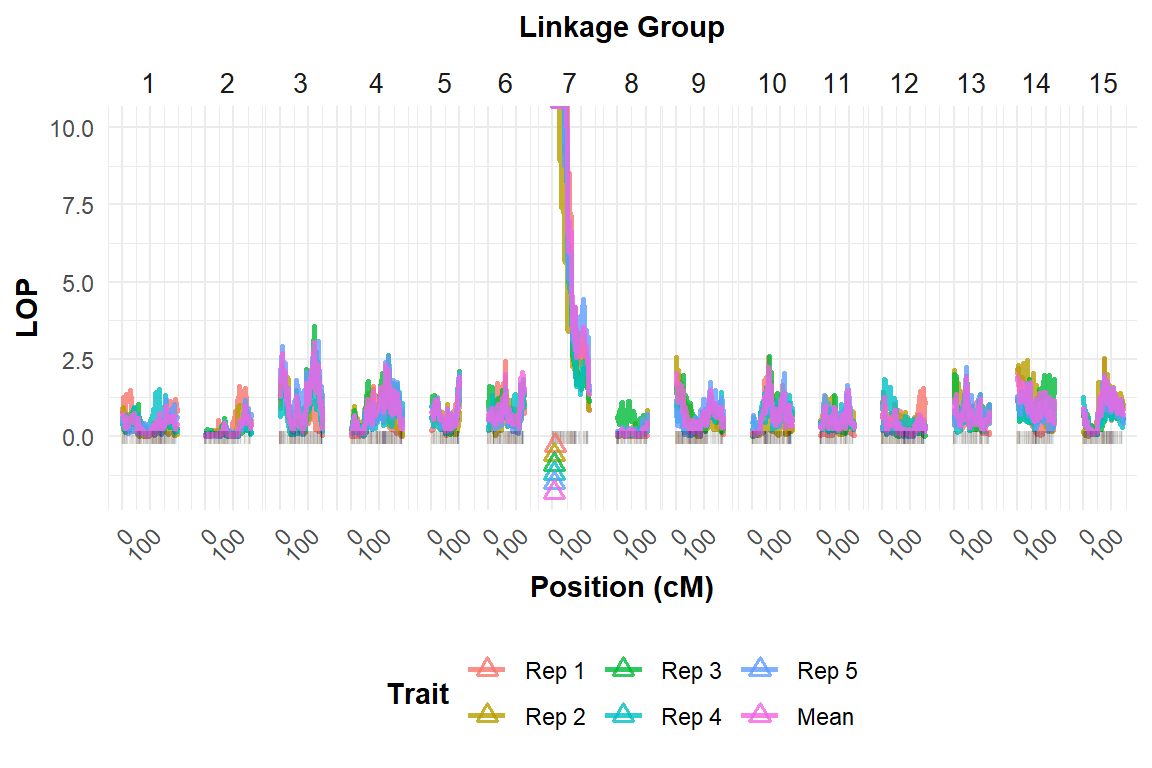
 Supplemental Figure S4. QTL profile for root-knot counts in the TB population. QTL mapping for the raw root-knot count data in reps 1 through 5 is shown together with the adjusted mean profile. LOP is the logarithm of *P*-values of score tests carried out every centimorgan (cM). The major QTL identified on LG 7 was present in all reps.


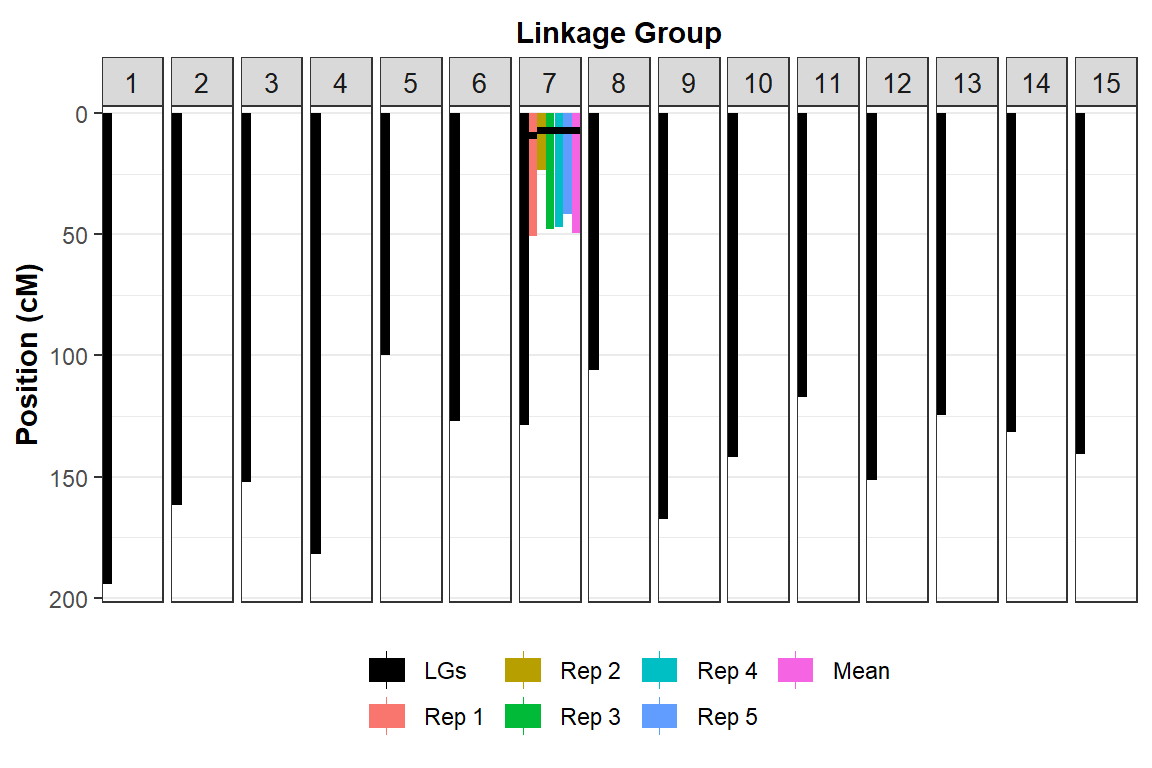


Supplemental Figure S5. Support interval (~95%) for the mapped location of QTL for root-knot counts in the TB mapping population on the raw data for reps 1 through 5 and their jointly adjusted mean.

Supplemental Table S1. Summary of Haley-Knott regression for haplotypes *h* and *i* for SNP S7_1038803. *i.h* represent the interaction between alleles *h* and *i*.

|  | Estimated Coefficients | Standard error | $t$ value | $P$(>\|$t$\|) |
| --- | --- | --- | --- | --- |
| μ | 50.7 | 2.475 | 20.5 | <2.22e-16 |
| *h* | –49 .0 | 4.124 | -11.9 | <2.22e-16 |
| *i* | –49.3 | 4.201 | -11.7 | <2.22e-16 |
| *i.h* | 50.0 | 6.239 | 8.0 | 5.46e-14 |
